# 8.E. Workshop: Climate crisis and public health: public health practitioners are key to successful litigation

**DOI:** 10.1093/eurpub/ckac129.495

**Published:** 2022-10-25

**Authors:** Organised by: EUPHA-LAW, EUPHA-ENV, Faculty of Public Health (UK), Groningen Centre for Health Law (Netherlands), Chair persons: Amandine Garde (EUPHA-LAW), Vlatka Matkovic (Belgium)

## Abstract

Encouraged by wins on asbestos and against Big Tobacco, the public health community and environmental advocates are turning to litigation to sue governments and private sector interests for public health harms from environmental pollution and climate change. Legal systems are as varied as health systems, yet there are principles common to all jurisdictions. In particular, the choice of legal forum is key: which court is best placed to hear a given case? Questions also arise regarding legal ‘standing’ - who can initiate legal proceedings? What is the most suitable legal basis for the claim? - it could be under a national constitutional protection of the right to life, a human rights treaty, or environmental or tort law. And, what evidence is required and what evidential standard should be met? Since 2020, four cases have been filed with the European Court of Human Rights in Strasbourg on States’ responsibilities for the physical or psychological impacts of climate change on human health. Moreover, in May 2021, the Dutch District Court in The Hague ordered Shell to reduce CO2 emissions by 45% by 2030, globally. To reflect on these developments, in October 2021 EUPHA co-hosted a webinar on public health, climate change and strategic litigation which highlighted how strategic partnerships between public health practitioners, environmental advocates, legal experts and affected communities can win cases, raise public awareness and push governments to act. In March 2022 EUPHA-LAW co-hosted a webinar on climate change litigation at the European Court of Human Rights. Sound scientific evidence is as critical to successful litigation as to effective public health policies. Increasingly, public health practitioners are asked to testify in court about the known health impacts of environmental harms. Collecting this evidence requires foresight, meticulous record-keeping, peer-support, and the courage to withstand questioning of professional capacity. In response to this need the Faculty of Public Health (UK), EUPHA-LAW, the Groningen Centre for Health Law in collaboration with Lancet Countdown, will publish a training package and toolkit for public health practitioners on public interest litigation to address climate change and environmental pollution. The workshop will review the role of litigation in advancing public health, and the outline and proposed uses for the toolkit, building on collaboration with environmental and legal organizations such as Greenpeace International and ClientEarth. Opportunities to engage with public health practitioners in regions outside Europe will also be explored.

**Key messages:**

• Strategic alliances between public health practitioners, environmental advocates, legal experts and affected communities can use litigation and legal process to address common goals.

• Public health practitioners are increasingly asked to testify in court about the known health impacts of environmental harms. Relevant knowledge and skills must be strengthened.

**Speakers/Panellists:**

**David Patterson**

University of Groningen, Groningen, Netherlands

**Farhang Tahzib**

Faculty of Public Health, Haywards Heath, UK

**Anniek de Ruijter**

University of Amsterdam, Amsterdam, Netherlands

**Scott Burris**

Center for Public Health Law Research, Temple University Beasley School of Law, Philadelphia, USA

